# CD4^+^ T cell subset imbalance contributes to diabetic kidney disease progression: immunopathological mechanisms and targeted therapeutic strategies

**DOI:** 10.3389/fimmu.2026.1882303

**Published:** 2026-09-17

**Authors:** Dingchang Zheng, Lingfei Zhang, Hang Li, Deping Wang, Caihong Jiao, Xiaolong Li, Songbo Fu

**Affiliations:** 1 CuiYing Honors College, Lanzhou University, Lanzhou, Gansu, China; 2 The First Clinical Medical College, Lanzhou University, Lanzhou, Gansu, China; 3 The Second Clinical Medical College, Lanzhou University, Lanzhou, Gansu, China; 4 Longnan First People’s Hospital, Longnan, Gansu, China; 5 Zhangye Second People’s Hospital, Zhangye, Gansu, China; 6 Department of Endocrinology, First Hospital of Lanzhou University, Lanzhou, Gansu, China; 7 Gansu Provincial Clinical Research Center for Endocrine Disease, Lanzhou, Gansu, China

**Keywords:** CD4+ T cells, diabetic kidney disease, immune metabolism, immunotargeted therapy, precision medicine, renal fibrosis, renal inflammation, T-cell subset balance

## Abstract

Diabetic kidney disease (DKD) is one of the most common microvascular complications of diabetes and a leading cause of end-stage renal disease. Recently, immune and inflammatory mechanisms have been recognized as critical in the pathogenesis of DKD, among which CD4^+^ T cell subset imbalance serves as a key contributor to renal inflammation and fibrosis. This narrative review synthesizes evidence from PubMed, Web of Science, and EMBASE (inception to March 2026) to provide a comprehensive overview of the immunopathological roles and molecular regulatory networks of major CD4^+^ T cell subsets, including T helper type 1, T helper type 2, T helper type 17 and regulatory T cells, in mediating renal injury through signature cytokines and signaling pathways. We also discuss the regulatory effects of hyperglycemia, hypoxia and lipid disorders on CD4^+^ T cell function, as well as the crosstalk between renal resident cells and CD4^+^ T cells via pathways such as programmed death-1/programmed death ligand-1 and nuclear factor-kappa B in amplifying inflammatory responses. Moreover, we outline current therapeutic strategies targeting CD4^+^ T cells, including traditional Chinese herbs, biologic agents, nonsteroidal mineralocorticoid receptor antagonists, sodium-glucose cotransporter 2 inhibitors and stem cell therapy, and evaluate their potential and limitations in balancing T cell subsets and alleviating renal injury. Although these approaches show promise, translating them into precise immunotherapy for DKD will require deeper mechanistic insight and rigorous clinical validation.

## 1 Introduction

Diabetic kidney disease (DKD), the most prevalent microvascular complication of diabetes mellitus, is a leading cause of end-stage renal disease (1). With the rising global prevalence of diabetes mellitus, the total number of affected individuals is projected to exceed 783 million by 2045 (2). The consequent potential surge in DKD may pose a severe challenge to the global public health system. Clinical diagnosis and staging of DKD largely depend on characteristic alterations in renal function and structure (3). Functionally, DKD is mainly characterized by impaired renal filtration and excretion, manifested as decreased estimated glomerular filtration rate (eGFR), increased urinary albumin excretion, and azotemia. Structurally, it exhibits typical renal tissue remodeling, including diffuse or nodular thickening of the glomerular basement membrane, mesangial matrix expansion, podocyte injury, and inflammatory cell infiltration in the renal interstitium. Accumulating evidence indicates that multiple mechanisms, including glucolipid metabolism disorders, hemodynamic abnormalities, genetic susceptibility, and immune-inflammatory responses, play critical roles in the initiation and progression of DKD (4–6). Currently, strict glycemic control remains the foundation of clinical management for DKD (7); novel targeted agents such as sodium-glucose cotransporter 2 inhibitors (SGLT2i), glucagon-like peptide-1 receptor agonists (GLP-1RAs), and finerenone have demonstrated definite efficacy in the clinical treatment of DKD (8–10). However, relying solely on conventional hypoglycemic and organ-protective strategies fails to completely reverse inflammatory injury and disease progression in DKD. Therefore, exploring the core targets of immune-inflammatory regulation and deciphering the immunomolecular mechanisms underlying DKD progression have become pivotal directions for breaking through the current therapeutic bottlenecks.

CD4^+^ T cells, as a major subset of T lymphocytes, serve as a pivotal regulator in immune responses and inflammatory modulation by undergoing directional differentiation and secreting specific cytokines (11). They mainly comprise helper T cells (Th, including functional subsets such as Th1, Th2, and Th17) and regulatory T (Treg) cells, which execute distinct functions in maintaining host immune homeostasis. Recent studies have demonstrated that the infiltration density of CD4^+^ T cells in the renal interstitium is significantly positively correlated with the severity of renal function deterioration; more importantly, the imbalanced polarization of their dominant cell subsets (particularly the typical Th1/Th2 and Th17/Treg imbalances) serves as the core driving force for triggering and amplifying local inflammatory responses in DKD (12–15). This subset shift directly disrupts the homeostatic balance between pro-inflammatory and anti-inflammatory cytokines, thereby triggering immune disorders and cascading inflammatory responses. Meanwhile, metabolic stressors such as hypoxia, hyperglycemia and hyperosmolarity, in conjunction with local innate immunity and cascading inflammatory responses, can further reshape the phenotypic characteristics and chemotactic infiltration capacity of CD4^+^ T cells (16, 17). Although these studies have preliminarily delineated the pathogenic role of CD4^+^ T cells in DKD progression, the spatiotemporal dynamics and the intricate regulatory crosstalk between subsets within the complex microenvironment remain incompletely understood. In addition, although novel agents such as SGLT2i have exhibited preliminary immunomodulatory potential, precise immunotherapy targeting CD4^+^ T cells is still in its infancy. Therefore, deeply dissecting the CD4^+^ T cell-mediated immune network and its precise mechanisms underlying DKD progression, and further developing individualized precise intervention strategies targeting this axis, represents an urgent need in current basic and translational research of DKD.

In this narrative review, we summarize the pathogenic mechanisms and molecular pathways underlying the involvement of distinct CD4^+^ T cell subsets in the initiation and progression of DKD. Furthermore, we highlight the crosstalk mechanisms between these subsets, glucolipid metabolism disorders and the renal resident cell microenvironment, aiming to delineate the complex immune-inflammatory network underlying CD4^+^ T cell-mediated synergistic regulation of multiple pathological factors in DKD. Finally, this review summarizes the emerging intervention strategies for DKD targeting CD4^+^ T cells as potential therapeutic targets, with the aim of providing theoretical support for future precision medicine translation.

### 1.1 Search strategy and selection criteria

This narrative review was based on a comprehensive literature search conducted in the PubMed, Web of Science, and EMBASE databases from their inception to March 2026. The search used combinations of the following keywords: “diabetic kidney disease” OR “diabetic nephropathy”; “CD4^+^ T cells” OR “T helper cells”; “Th1,” “Th2,” “Th17,” “Treg”; “immune imbalance”; “inflammation”; “fibrosis”; “SGLT2 inhibitor”; “stem cell therapy”; and “immunotherapy.” To avoid bias, we prioritized original research articles (*in vitro*, *in vivo*, and clinical studies) and comprehensive reviews published in English. Two authors independently screened titles and abstracts for relevance, focusing on the immunopathological roles of CD4^+^ T cell subsets in DKD progression and therapeutic strategies targeting these cells. The final set of references was selected by consensus among all authors. No formal quality assessment tool was applied.

## 2 CD4^+^ T cell subsets and their critical functions in DKD

Inflammatory responses persist throughout the entire pathological progression of DKD. Infiltrating inflammatory cells and their secreted cytokines not only disrupt insulin signaling and exacerbate insulin resistance, but also directly drive renal tissue fibrosis and renal function decline (15, 18, 19). Clinical evidence indicates that the composition of CD4^+^ T cell subsets in the peripheral blood of patients with DKD has shifted markedly (**Figure 1**): the proportions of pro-inflammatory subsets typified by Th1 and Th17 cells are significantly elevated, whereas the anti-inflammatory phenotypes of Th2 and Treg cells are sharply diminished (20, 21). Consistent with this, studies in DKD mouse models have observed increased renal infiltration of Th1/Th17 cells and decreased Th2/Treg proportions, with the Th17/Treg ratio showing a significant positive correlation with key markers of renal injury, including the urinary albumin/creatinine ratio (UACR) (15, 22, 23). Bioinformatic analysis and experimental verification collectively demonstrate that the dual immune imbalance of Th1/Th2 and Th17/Treg arises from the synergistic disruption of epigenetic and transcriptional regulatory networks driven by hyperglycemia, which directly induces glomerulosclerosis and tubulointerstitial injury by disrupting the dynamic balance between pro- and anti-inflammatory networks (15, 23, 24). Specifically, the expression of Th1-related long non-coding RNAs (lncRNAs), such as *IFNG-AS1* and *FGD5-AS1*, and their transcription factor T-box transcription factor 21 (*TBX21*), is upregulated, whereas the expression of the Th2-related lncRNA *TH2LCRR* and its transcription factor GATA-binding protein 3 (*GATA3*) is downregulated, resulting in enhanced pro-inflammatory Th1 responses and weakened anti-inflammatory Th2 responses. Meanwhile, the expression of the Th17-related lncRNA *CRNDE* and its transcription factor retinoic acid-related orphan receptor C (*RORC*) is increased, promoting IL-17 secretion, whereas the expression of the Treg-related lncRNA *SNHG1* and its transcription factor forkhead box protein 3 (*FOXP3*) is decreased, leading to impaired immunosuppressive function. Together, these events ultimately constitute a dual vicious cycle characterized by excessive activation of pro-inflammatory pathways and insufficient activity of anti-inflammatory pathways. These findings indicate that the magnitude of CD4^+^ T cell subset skewing may serve as a potential biomarker for assessing the progression of renal injury in DKD. Herein, we discuss the pathogenic and regulatory mechanisms of each key subset during DKD progression.

**Figure 1:**
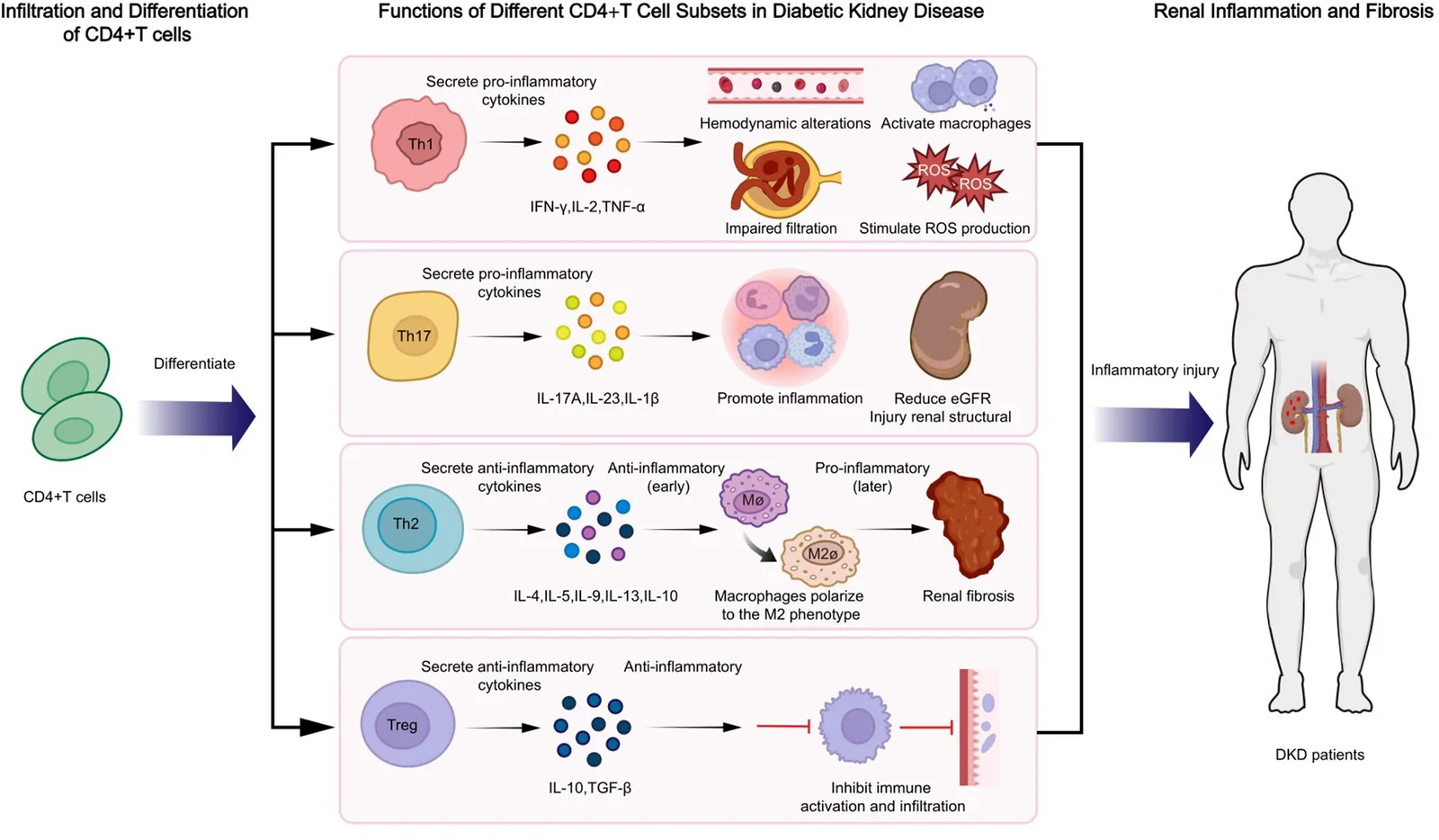
Imbalance pattern of CD4^+^ T cell subsets in diabetic kidney disease (DKD). In DKD microenvironment, the differentiation ratio and tissue infiltration abundance of CD4^+^ T lymphocytes are markedly disrupted. Pro-inflammatory T helper type 1 (Th1) and T helper type 17 (Th17) subsets expand substantially, accompanied by elevated secretion of signature pro-inflammatory cytokines including interferon-γ (IFN-γ), tumor necrosis factor-α (TNF-α), and interleukin-17A (IL-17A). Conversely, anti-inflammatory T helper type 2 (Th2) and regulatory T (Treg) cell populations are significantly depleted, with reduced production of protective cytokines interleukin-4 (IL-4), interleukin-10 (IL-10), and transforming growth factor-β (TGF-β). The dual imbalance of Th1/Th2 and Th17/Treg axes constitutes the core immunopathological hallmark of DKD, which continuously amplifies intrarenal inflammatory cascades and drives progressive renal fibrosis. DKD, diabetic kidney disease; Th1, T helper type 1; Th2, T helper type 2; Th17, T helper type 17; Treg, regulatory T cell; IFN-γ, interferon-γ; TNF-α, tumor necrosis factor-α; IL, interleukin; TGF-β, transforming growth factor-β.

### 2.1 Th1 cells

Th1 cells, as the prototypical pro-inflammatory subset, primarily mediate immune and inflammatory responses by secreting effector cytokines including interferon-γ (IFN-γ), interleukin-2 (IL-2), and tumor necrosis factor-α (TNF-α) (25). At the level of differentiation regulation, serum IL-12 levels are significantly elevated in patients with DKD and closely correlated with eGFR decline, while *IFNG-AS1* is upregulated in peripheral blood and positively associated with *TBX21* mRNA levels and indicators of deteriorating renal function (15, 20, 23, 24). These clinical findings suggest that elevated IL-12 and aberrant activation of the *IFNG-AS1*/*TBX21* axis may contribute to Th1 skewing, thereby exacerbating inflammatory injury in DKD. In terms of chemotactic recruitment, the directed migration of Th1 cells to the renal lesion site relies on local chemokine concentration gradients. Clinical evidence indicates that C-X-C motif chemokine ligand 9 (CXCL9) levels in the serum and urine of DKD patients are significantly higher than those in healthy controls (26). *In vitro* studies further revealed that advanced glycation end products (AGEs) upregulate the expression of CXCL9 and its receptor C-X-C chemokine receptor type 3 (CXCR3) in mouse podocytes, activate the Janus kinase 2/signal transducer and activator of transcription 3 (JAK2/STAT3) signaling pathway, and induce podocyte apoptosis and inflammation (26). Animal experiments have demonstrated that the extracellular matrix component biglycan acts as an endogenous danger signal, inducing CXCL10 secretion in macrophages (Mø) via the Toll-like receptor 4/TIR-domain-containing adapter-inducing interferon-β (TLR4/TRIF) signaling pathway and promoting CXCL9 release in synergy with IFN-γ; together, these chemokines recruit CXCR3-positive Th1 and Th17 cells to infiltrate the kidney, thereby aggravating proteinuria (27). Concurrently, this same study observed a positive correlation between plasma biglycan levels and CXCL9/CXCL10 levels in DKD patients, providing clinical correlative evidence for the proposed mechanism (27). In addition, the binding of C-C motif chemokine ligand 5 (CCL5) to its receptor C-C chemokine receptor type 5 (CCR5) represents another important pathway that guides the directional migration of these cells (28).

The IFN-γ and TNF-α released by Th1 cells after infiltration can mediate renal tissue damage through multiple mechanisms. Clinical studies have demonstrated a positive correlation between serum IFN-γ and TNF-α levels and urinary albumin excretion rate in DKD patients, and both cytokines have been recognized as potential molecular markers for evaluating disease severity in DKD (13). *In vitro* experiments have shown that AGEs markedly enhance the capacity of activated T cells to produce IFN-γ (29). Consistent with this, animal studies have confirmed massive macrophage infiltration and elevated expression of pro-inflammatory and pro-fibrotic factors in the kidneys of DKD rats (30). Among these factors, TNF-α not only alters glomerular hemodynamics and reduces filtration rate, but also directly induces podocyte and tubular cell toxicity, triggers local reactive oxygen species generation, and synergizes with transforming growth factor-beta (TGF-β) to indirectly promote renal inflammation and fibrosis (31). Notably, IFN-γ and TNF-α can further recruit and activate Mø, establishing a pro-inflammatory positive feedback loop that amplifies the local inflammatory cascade; however, the specific signaling pathways within this loop remain to be experimentally validated (30). Thus, excessive infiltration of Th1 cells and the ensuing pro-inflammatory cytokine storm represent one of the core drivers underlying the progression of early-stage DKD lesions. Nevertheless, the immune network does not operate unidirectionally, and Th2 cells, which antagonize Th1 cells, also play an indispensable role in the pathogenesis of DKD.

### 2.2 Th2 cells

As the natural antagonist of Th1 cells, Th2 cells constitute the major anti-inflammatory subset within the CD4^+^ T-cell population. They not only initiate and sustain humoral immune responses but also directly suppress the activation of Th1 cells and limit the propagation of inflammatory reactions (32). Clinical cross-sectional studies have demonstrated that Th2 cell function is frequently and markedly suppressed in patients with DKD, with their counts showing a significant negative correlation with urinary protein levels (2).

Clinical evidence from DKD patients further indicates that Th2 cells orchestrate a sophisticated immunoregulatory network by secreting cytokines including IL-4, IL-5, IL-9, IL-10, and IL-13 (13). Among these, the core factor IL-4 not only regulates cell proliferation and inflammatory cytokine secretion but also activates the STAT6 signaling pathway. Early studies using *Stat6*-deficient mice established that IL-4-induced STAT6 activation is a key event for Th2 cell differentiation and *GATA3* upregulation (33–35). Subsequently, Zheng and Flavell, employing retroviral overexpression and antisense oligonucleotide techniques in mouse CD4^+^ T cells *in vitro*, demonstrated that *GATA3* is both necessary and sufficient to drive Th2 cytokine gene expression (36). Meanwhile, *in vitro* experiments have shown that IL-4 upregulates the expression of the IL-4 receptor α-chain (IL-4Rα) on the surface of Th2 cells, forming an IL-4/IL-4Rα positive feedback loop that enhances the responsiveness of Th2 cells to IL-4 (37). Furthermore, *in vitro* studies collectively revealed that IL-2, produced by activated T cells, activates the STAT5 pathway and cooperates with *GATA3* to drive sustained Th2 cytokine production, which is critical for Th2 differentiation (38, 39). It should be noted that all of the above positive feedback regulatory mechanisms have been established in mouse T cell *in vitro* differentiation systems, and direct experimental validation in the DKD renal microenvironment is currently lacking.

However, the effects of Th2 cell-derived cytokines are not universally beneficial, with IL-13 serving as a notable example. In patients with DKD, serum IL-13 levels decline significantly as nephropathy progresses, suggesting that this cytokine may be consumed or suppressed under disease conditions (20). *In vitro* experiments have shown that recombinant IL-4 and IL-13 can directly act on human renal tubular epithelial cells (RTCs) to upregulate the expression of TGF-β1, fibronectin, and type I collagen; furthermore, under high glucose conditions, both IL-4 and IL-13 synergize with hyperglycemia to markedly enhance the production of these pro-fibrotic factors (40). From a dose-response perspective, IL-4 upregulates TGF-β1 and fibronectin in HK-2 cells in a concentration- and time-dependent manner (40). Collectively, these findings suggest that, within the chronic hyperglycemic microenvironment of DKD, persistently present IL-4 and IL-13 may shift from protective factors to pro-fibrotic mediators, although this hypothesis still requires further validation in *in vivo* models and human DKD kidney tissues. The roles of other Th2-derived cytokines in DKD are also complex. Clinical studies have demonstrated that serum IL-9 levels in patients with DKD are significantly lower than those in patients with uncomplicated type 2 diabetes (T2D), yet positively correlated with urea and microalbuminuria, suggesting that these changes are associated with the severity of renal impairment rather than reflecting simple compensatory depletion (41). In animal experiments, triptolide has been shown to ameliorate renal injury by upregulating the decreased Th2 cytokine IL-10 and correcting the Th1/Th2 imbalance in diabetic rats; moreover, such anti-inflammatory and protective effects of IL-10 have been validated in multiple kidney disease models (30). Basic research has further demonstrated that IL-10 and IL-4 can synergistically inhibit IFN-γ secretion, thereby attenuating Th1-mediated renal injury, which constitutes a key cellular mechanism underlying the protective effects of Th2 cells (42, 43). Taken together, these observations suggest that in the early stages of DKD, Th2 cells may limit inflammation primarily through the secretion of cytokines such as IL-10; however, in later stages, their persistent activation may contribute to the progression of fibrosis. Future studies are warranted to validate these findings, which have been derived mainly from animal and *in vitro* experiments, in human DKD. Beyond the canonical Th1/Th2 axis, dysregulation of the Th17/Treg balance has emerged as a major focus in this field for its role in the progression of DKD.

### 2.3 Th17 cells

Th17 cells represent another key pro-inflammatory subset of CD4^+^ T cells, and their pathogenic roles in diverse chronic inflammatory diseases have attracted considerable attention in recent years (44). Studies have shown that the proportion of Th17 cells is markedly elevated in both the peripheral blood and renal tissue of patients with DKD, and this increase correlates positively with UACR. This is accompanied by high expression of signature pro-inflammatory cytokines such as IL-17A (45). These findings solidify the central role of Th17 cells in the pathogenesis of DKD, and their activation status has emerged as an independent indicator of disease severity (21, 46).

The differentiation of Th17 cells and the release of their effector cytokines are governed by a sophisticated molecular signaling network (**Figure 2**). In diabetic animal models, multiple studies have revealed that the cascade composed of serum/glucocorticoid-regulated kinase 1 (SGK1), FoxO1, and RORγt plays a central regulatory role (47, 48). Specifically, the hyperglycemic environment upregulates renal SGK1 expression; activated SGK1 subsequently phosphorylates and inactivates the transcription factor FoxO1, thereby relieving FoxO1-mediated repression of the *Rorc* promoter and ultimately enhancing RORγt transcriptional activity (46, 49). *In vitro* studies have further confirmed that RORγt is a key transcription factor driving Th17 cell differentiation, and activation of this pathway not only promotes IL-23 receptor expression to stabilize the Th17 phenotype but also triggers the release of pro-inflammatory cytokines such as IL-17A (49, 50). However, the activation status and clinical significance of this pathway in human DKD remain to be further investigated.

**Figure 2:**
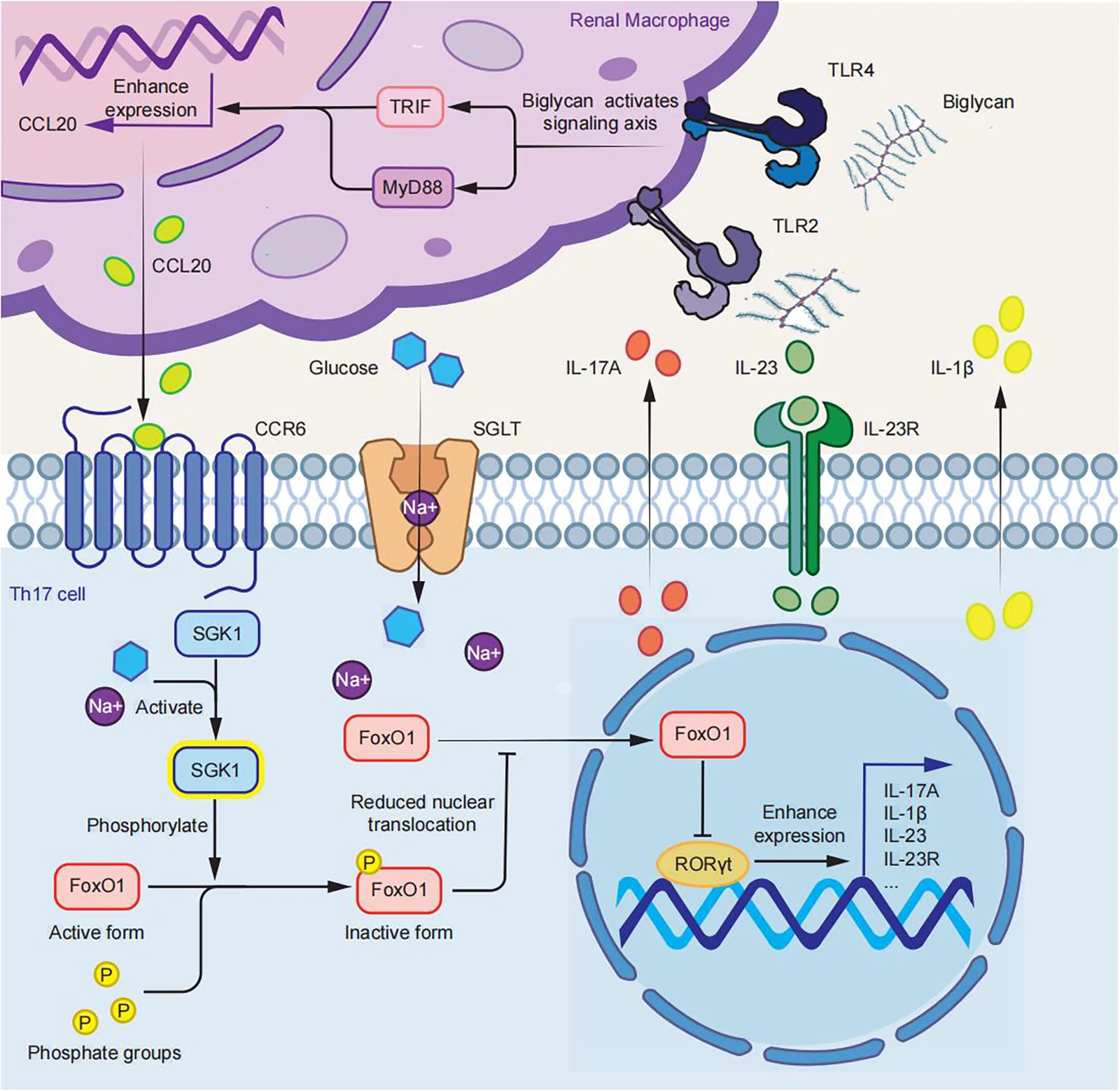
Molecular regulatory network of Th17 cell differentiation and activation in DKD. Persistent hyperglycemia abnormally activates the serum/glucocorticoid-regulated kinase 1 (SGK1)/phosphorylated forkhead box O1 (p-FoxO1)/retinoid-related orphan receptor γt (RORγt) signaling cascade. Activated RORγt transcriptionally upregulates interleukin-23 receptor (IL-23R) expression to stabilize Th17 lineage commitment. Additional upstream regulators including microRNA-155, TGF-β, and IL-6 synergistically modulate Th17 differentiation. Mature Th17 cells secrete large quantities of IL-17A and IL-1β, which act as primary effector mediators to trigger renal inflammatory injury and extracellular matrix deposition. DKD, diabetic kidney disease; SGK1, serum/glucocorticoid-regulated kinase 1; p-FoxO1, phosphorylated forkhead box O1; RORγt, retinoid-related orphan receptor γt; IL-23R, interleukin-23 receptor; miR-155, microRNA-155; TGF-β, transforming growth factor-β; IL, interleukin.

Beyond hyperglycemia, high extracellular sodium represents another critical upstream activator of SGK1. SGLT2, a sodium-glucose cotransporter expressed on RTCs, is upregulated by high glucose in DKD, thereby enhancing Na^+^ reabsorption and increasing the local sodium load. *In vitro* experiments by Wang et al. showed that high sodium promotes CD4^+^ T cell differentiation toward Th17 cells in a concentration-dependent manner and upregulates the SGK1/p-FoxO1/IL-23R pathway (47). Notably, conditioned medium from RTCs with SGLT2 knockdown exhibited a markedly reduced capacity to induce Th17 differentiation, suggesting that SGLT2 can indirectly influence T cell fate by modulating sodium ion concentrations. *In vivo* studies further confirmed that db/db mice display elevated serum sodium levels, and that knockdown of either SGLT2 or SGK1 reduces serum sodium concentration and restores the Th17/Treg balance in both the kidney and peripheral blood (47, 51). It should be emphasized, however, that direct *in vivo* quantitative evidence demonstrating that increased SGLT2 activity can raise renal interstitial Na^+^ concentration to the threshold required for modulating CD4^+^ T cell differentiation is still lacking. Although ^23^Na-MRI studies have documented increased sodium content in tissues such as the skin of patients with T2D, pointing to systemic sodium dysregulation, precise measurement of local sodium concentration in the renal interstitium remains methodologically challenging. This gap represents a critical barrier linking *in vitro* mechanistic findings to *in vivo* pathophysiology (52). Furthermore, Th17 cell differentiation is also coordinately regulated by microRNA-155 (miR-155), RORα, interferon regulatory factor 4 (IRF4), and various microenvironmental cytokines such as TGF-β and IL-6, collectively shaping a complex regulatory landscape for cell fate determination (22, 53–55).

The directed migration of Th17 cells to renal lesions similarly depends on the chemokine network, which relies on the precise interplay between the chemokine receptors CCR6 and CXCR3 expressed on their surface and the corresponding ligands produced locally (26). Clinically, serum and urinary levels of CXCL9, a ligand for CXCR3, are significantly elevated in DKD patients, providing an important clinical clue for the chemotactic recruitment of Th17 cells to the kidney. At the mechanistic level, animal studies have uncovered multiple signaling pathways driving this process. One involves the extracellular matrix component biglycan, which is upregulated in the kidneys of diabetic or lupus nephritis mice. As a damage signal, biglycan activates local Mø through the TLR4/TRIF and TLR2/4/myeloid differentiation primary response 88 signaling axes, leading to the abundant secretion of various chemokines, including CCL20 (a CCR6 ligand) and CXCL9/10 (CXCR3 ligands), thereby precisely guiding the infiltration of Th1 and Th17 cells into renal tissue (27). Notably, the same study confirmed a positive correlation between plasma biglycan levels and CXCL9/CXCL10 levels in DKD and lupus nephritis patients, offering preliminary clinical correlative evidence for these animal model findings (27). In addition, a recent study in a mouse model of type 2 DKD found that highly expressed heat shock protein 70 (HSP70) in the kidney indirectly increases the Th17/Treg ratio by suppressing T-cell immunoglobulin and mucin domain-3 (Tim-3), thereby exacerbating local inflammation. This suggests that the local injury microenvironment may shape the pathogenic functions of Th17 cells through multiple pathways (56).

Infiltrating Th17 cells perpetually aggravate DKD progression via their key effector molecule IL-17A. In diabetic mouse models, blockade of this signaling axis with an IL-17A neutralizing antibody significantly halted the progression of proteinuria and ameliorated renal pathological injury, providing direct *in vivo* evidence for the pathogenic role of IL-17A (50). Observational clinical studies also suggest an association between circulating IL-17A levels and progressive eGFR decline in DKD patients, although these findings await validation in larger-scale clinical studies (57). Concurrently, IL-1β secreted by Th17 cells can also activate inflammatory pathways in renal resident cells through non-canonical signaling, thereby exacerbating tissue necrosis (58).

### 2.4 Treg cells

Treg cells function as a critical “brake system” that maintains immune tolerance and tissue homeostasis. In animal models of DKD, Treg cells exhibit reduced numbers and impaired immunosuppressive function (47). For example, in a mouse model of streptozotocin (STZ)-induced DKD, a decrease in the renal Th17/Treg ratio was closely associated with an improvement in the UACR, supporting the critical role of Treg cells in restraining renal inflammation during DKD progression (22). From a fundamental immunological perspective, Treg cells exert their immunosuppressive effects mainly through two pathways. First, via paracrine secretion of potent inhibitory cytokines such as IL-10 and TGF-β, they strongly suppress the activation of various effector T cells and NK cells, thereby limiting the chemotactic infiltration of immune cells into tissues; in animal models of DKD, upregulation of these inhibitory cytokines has been associated with attenuated renal inflammation and fibrosis, although direct *in vivo* evidence confirming their Treg cell origin remains limited (59–62). Second, Treg cells constitutively express high levels of cytotoxic T-lymphocyte-associated protein 4 (CTLA-4) on their surface, which binds to CD80/CD86 on antigen-presenting cells, thereby inhibiting their function in a cell contact-dependent manner (63). Studies in other tissue injury models have demonstrated that Treg cells can modulate inflammatory signaling in innate immune cells, such as Mø and microglia, through a CTLA-4-dependent contact mechanism (64). However, direct *in vivo* evidence for the role of CTLA-4-mediated Treg–innate immune cell interactions within the kidney in DKD is currently lacking. Collectively, these dual mechanisms constitute a critical defense line for maintaining immune homeostasis, and their dysfunction represents a key factor driving the progression of DKD.

From the perspective of Treg cell biology, *FOXP3* serves as the master transcription factor that determines Treg lineage identity and immunosuppressive function, and its expression is dynamically regulated by multiple microenvironmental cues—a regulatory mechanism that has been extensively validated in various experimental systems (63). In *in vitro* co-culture systems, M2ø can directly upregulate *Foxp3* expression in T cells and promote their differentiation into Treg cells. In db/db DKD mice, pharmacological interventions that promote M2ø polarization significantly increase the number of renal Treg cells and attenuate renal injury, suggesting that this M2–Treg regulatory axis may also exert protective effects *in vivo*, although the precise molecular mechanisms operating within the living kidney remain to be elucidated (65).

The functional stability of Treg cells is subject to negative regulation by multiple signals. In db/db diabetic mice, hyperglycemia and high sodium conditions activate SGK1 kinase, leading to an imbalance in the Th17/Treg ratio. SGK1 serves not only as a positive driver of Th17 cell differentiation but also as a negative regulatory node for Treg cell function. Mechanistically, SGK1 phosphorylates FoxO1 and promotes its nuclear export, thereby suppressing *FOXP3* transcription and directly compromising Treg lineage stability and immunosuppressive capacity. Under high sodium conditions, persistently elevated SGK1 activity further biases T cell fate toward Th17 differentiation while antagonizing Treg maintenance. Moreover, high salt intake increases reactive oxygen species production, which further activates SGK1, exacerbating Treg loss and Th17 expansion (47, 66). Treg cell maintenance is also actively antagonized by local injury signals. In type 2 DKD mice, high renal expression of HSP70 impairs Treg function by suppressing Tim-3 (56). Furthermore, *in vitro* and animal studies indicate that hypoxic stress can transiently increase Treg abundance to counteract inflammation through hypoxia-inducible factor 1-alpha (HIF-1α)-mediated transcriptional activation of *Foxp3*; however, whether this compensatory pathway confers significant protection in the DKD kidney remains to be validated (67). Notably, the consequences of Treg loss extend far beyond the kidney. Clinical studies have shown that Treg cells are markedly reduced in visceral adipose tissue of obese insulin-resistant patients (68). Experiments in db/db mice further confirmed that Treg depletion exacerbates insulin resistance and renal injury, whereas adoptive transfer of Treg cells ameliorates both (68). Thus, when pathogenic factors overwhelm compensatory mechanisms and lead to Treg loss, not only does the local anti-inflammatory defense in the kidney collapse, but systemic insulin resistance may also be aggravated through peripheral pathways, creating a vicious cycle that drives disease progression.

## 3 CD4^+^ T cell-regulated factors and key molecular pathways in DKD

### 3.1 Regulatory effects of metabolic factors on CD4^+^ T cells in DKD

Metabolic disorders are the core pathological basis underlying the initiation and progression of DKD. Accumulating evidence indicates that hyperglycemia, dyslipidemia, and hypoxia not only directly injure renal parenchyma, but also remodel metabolic pathways in CD4^+^ T cells, thereby altering their tissue infiltration capacity and disrupting subset homeostasis (16, 17) (**Figure 3**). Such metabolic reprogramming-driven immune dysregulation further amplifies local inflammation, forming a vicious cycle of “metabolic dysfunction–immune dysregulation–renal injury” (47). Here, we focus on how three core metabolic factors—hyperglycemia, hypoxia, and lipid metabolic dysfunction—profoundly participate in and regulate this immunopathological process.

**Figure 3:**
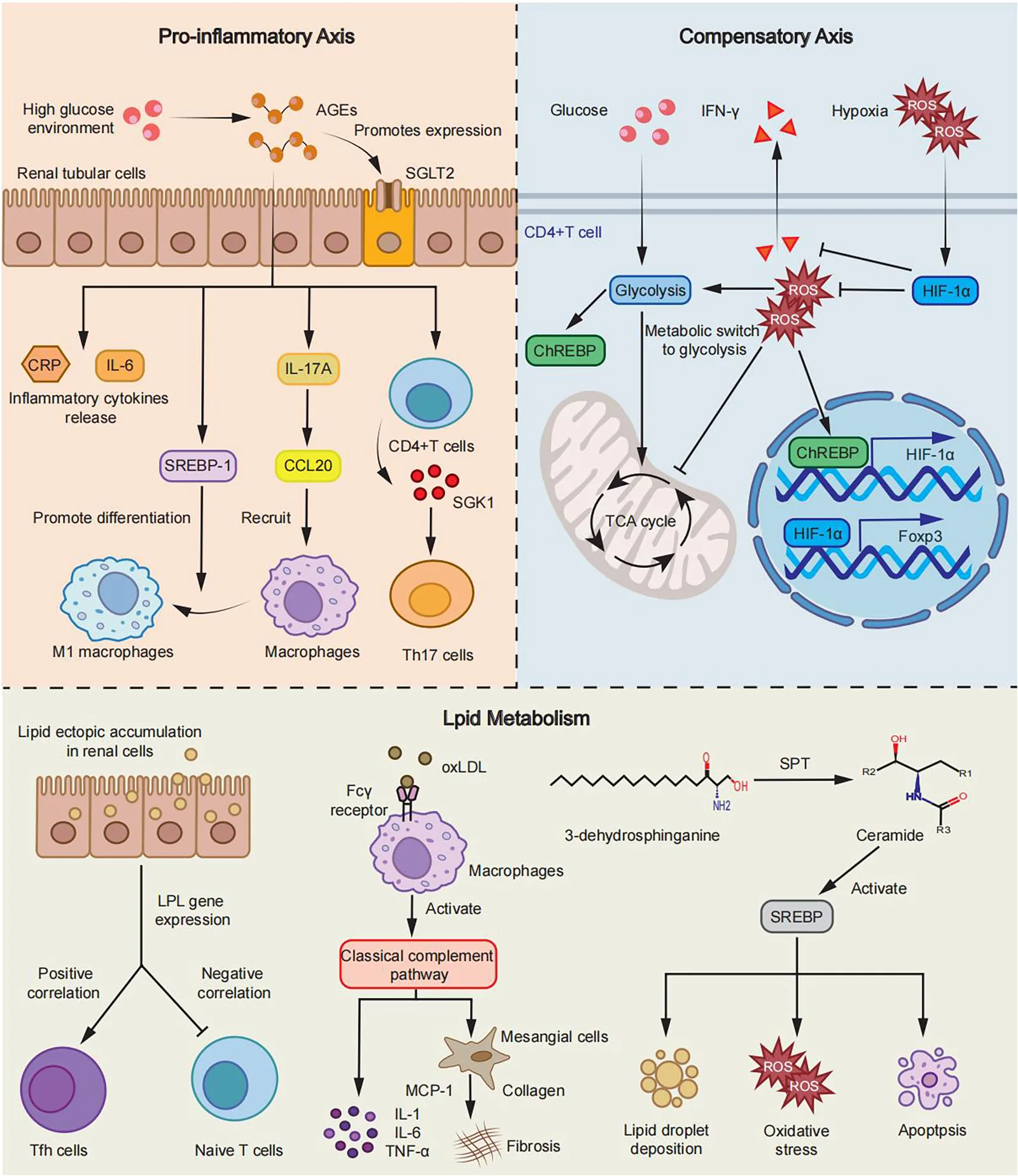
Regulatory mechanism of high glucose, hypoxia and lipid metabolism on CD4^+^ T cells in DKD. Hyperglycemia triggers abundant advanced glycation end products accumulation and reactive oxygen species generation, upregulates tubular sodium-glucose cotransporter 2 (SGLT2) to elevate interstitial sodium levels and activates the SGK1/p-FoxO1 cascade, which drives M1 macrophage polarization and Th17 differentiation to aggravate renal inflammation; meanwhile, high glucose initiates a compensatory protective pathway via carbohydrate response element-binding protein-dependent *HIF1A* transcription in CD4^+^ T cells to restrict excessive Th1 activation. Hypoxia stabilizes HIF-1α, which transcriptionally activates forkhead box protein 3 (*FOXP3*) to expand functional Treg cells and shifts CD4^+^ T cell energy metabolism from oxidative phosphorylation to glycolysis to suppress overactive T cell-mediated inflammation. Lipid metabolic dysfunction causes ectopic lipid deposition in podocytes and renal tubular epithelial cells to induce lipotoxic injury; lipoprotein lipase negatively correlates with naive CD4^+^ T cells and positively correlates with T follicular helper cells, while oxidized low-density lipoprotein, acetyl-CoA carboxylase 1 and retinoid-related orphan receptor γt (RORγt)-dependent cholesterol metabolites jointly disturb the Th17/Treg balance, collectively disrupting intrarenal immune homeostasis. DKD, diabetic kidney disease; AGEs, advanced glycation end products; ROS, reactive oxygen species; SGLT2, sodium-glucose cotransporter 2; SGK1, serum/glucocorticoid-regulated kinase 1; p-FoxO1, phosphorylated forkhead box O1; Th17, T helper type 17; ChREBP, carbohydrate response element-binding protein; HIF-1α, hypoxia-inducible factor-1α; Th1, T helper type 1; FOXP3, forkhead box protein 3; Treg, regulatory T cell; LPL, lipoprotein lipase; Tfh, T follicular helper cell; oxLDL, oxidized low-density lipoprotein; ACC1, acetyl-CoA carboxylase 1; RORγt, retinoid-related orphan receptor γt.

#### 3.1.1 Hyperglycemia and hypoxia

As a central contributor of DKD, hyperglycemia disrupts renal microenvironmental homeostasis primarily by inducing oxidative stress, triggering inflammatory responses, and accelerating tissue fibrosis (69, 70). During this process, hyperglycemia exerts a highly bidirectional regulatory effect on CD4^+^ T cells. With regard to pro-inflammatory effects, the abundant AGEs generated under high-glucose conditions not only directly induce the release of inflammatory factors such as C-reactive protein and IL-6, but also indirectly amplify the pro-inflammatory effect of CD4^+^ T cells by strengthening the “IL-17A-CCL20-Mø recruitment” axis and promoting Mø polarization toward the M1 phenotype via sterol regulatory element-binding protein 1 (71–73). In the db/db diabetic mouse model, hyperglycemia also upregulates SGLT2 expression in RTCs and, by increasing local sodium concentrations, activates the SGK1/p-FoxO1/IL-23R signaling axis in CD4^+^ T cells, thereby directly driving Th17/Treg imbalance and accelerating DKD progression (47). In terms of compensatory anti-inflammatory mechanisms, a recent study using T cell-specific HIF-1α knockout mice has uncovered an important self-protective pathway in CD4^+^ T cells: under high glucose conditions, the glucose-responsive transcription factor carbohydrate response element-binding protein (ChREBP) directly binds to the *Hif-1α* promoter and drives its expression; upregulated HIF-1α then attenuates DKD progression, at least in part, by limiting IFN-γ secretion from Th1 cells (74). Notably, through *in vivo* approaches including bone marrow transplantation and T cell adoptive transfer, this study demonstrated that the protective role of HIF-1α in T cells is both specific and essential in the context of DKD (74). This sophisticated bidirectional regulation essentially reflects the dynamic interplay between “pro-inflammatory injury” and “compensatory protection” in the body under metabolic stress.

As another key pathophysiological process in DKD progression, hypoxia mainly arises from the imbalance between increased renal oxygen consumption and insufficient local blood perfusion, which markedly induces the synthesis and activation of HIF-1α in renal tissue (75). In terms of renal protection, overexpressed HIF-1α in renal tissue delays pathological progression of DKD by reducing cellular reactive oxygen species production, inhibiting apoptosis of renal resident cells, and alleviating oxidative stress injury in renal tissue (76). Meanwhile, HIF-1α acts directly on CD4^+^ T cell subsets. It not only reduces the total number of CD4^+^ T cells by inhibiting signaling pathways related to cell proliferation, but also transcriptionally induces *FOXP3* expression by directly binding to the promoter region of *FOXP3*—the signature transcription factor of Treg cells. This increases the abundance and anti-inflammatory function of Treg cells, which in turn restricts local renal inflammation and maintains immune homeostasis through their intrinsic immunosuppressive activity (77, 78). The HIF-1α–Treg regulatory pathway described above has been validated in basic immunological studies; however, its direct role in the renal microenvironment of DKD remains to be further investigated. Furthermore, under hypoxic tension in DKD renal tissue, CD4^+^ T cells undergo metabolic pathway reprogramming, shifting their energy metabolism from oxidative phosphorylation to glycolysis. This glycolytic metabolic phenotype suppresses CD4^+^ T cell proliferation and induces an immunosuppressive state, thereby attenuating renal injury caused by excessive immune activation (73). Collectively, hypoxia intricately links energy metabolic status to immune function by inducing metabolic reprogramming in CD4^+^ T cells and directly regulating Treg cells. This provides a novel perspective for understanding how “the hypoxia–HIF-1α” axis maintains immune homeostasis in DKD.

Of note, studies using T cell-specific knockout mouse models have demonstrated that hyperglycemia upregulates HIF-1α expression in CD4^+^ T cells in a ChREBP-dependent manner, thereby limiting pathogenic Th1 cell responses and attenuating renal injury in DKD (74). Given that hyperglycemia and hypoxia frequently coexist in the pathological microenvironment of DKD, it is plausible that they may synergistically reinforce HIF-1α transcriptional signaling in CD4^+^ T cells, together constituting an endogenous protective barrier that restrains the renal inflammatory cascade. Therefore, future investigations should move beyond the isolated analysis of individual factors and instead focus on dissecting the interplay between hyperglycemia and hypoxia, which represents a pivotal breakthrough direction for elucidating the complex roles of CD4^+^ T cells in DKD.

#### 3.1.2 Lipid metabolic dysfunction

Lipid metabolic dysfunction plays a pivotal pathogenic role in DKD, characterized mainly by ectopic lipid accumulation in RTCs and podocytes. Such ectopic lipid deposition not only disrupts the structure and function of the glomerular filtration barrier via direct lipotoxicity but also activates intracellular inflammatory signaling pathways and triggers local inflammation, collectively leading to increased urinary albumin excretion and accelerated DKD progression (79, 80). More recently, the interplay between dysregulated lipid metabolism and CD4^+^ T cell immunoregulation has opened up new perspectives for understanding the pathogenesis of DKD.

Dong et al., through systematic bioinformatics analysis, identified the lipoprotein lipase gene (LPL) as a core pathogenic gene in DKD, involved in cholesterol metabolism, glycerolipid metabolism, and the peroxisome proliferator-activated receptor signaling pathway. Immunohistochemical validation in the same study further revealed that LPL protein expression in renal tissues of DKD patients decreased with increasing disease severity and was strongly negatively correlated with TNF-α. Moreover, bioinformatics analysis of immune cell infiltration based on transcriptomic data predicted that LPL expression levels were negatively correlated with the proportion of naïve CD4^+^ T cells and positively correlated with the proportion of T follicular helper (Tfh) cells (81). These findings suggest that lipid metabolic abnormalities may influence the differentiation and functional balance of CD4^+^ T cells by regulating key molecules such as LPL, thereby exacerbating renal inflammatory injury through immune dysregulation; however, this hypothesis still awaits direct causal validation in *in vitro* and *in vivo* experiments.

The regulation of CD4^+^ T cells by lipid metabolism in DKD may extend beyond modulation of LPL activity. At the animal model level, Han et al., using metabolomics, identified 3-dehydrosphinganine as a novel core biomarker; this molecule is converted to ceramide by serine palmitoyltransferase and promotes lipid droplet deposition, oxidative stress, and apoptosis through activation of *SREBP* genes, providing *in vivo* evidence for the pathogenic role of sphingolipid metabolism in DKD (82, 83). At the clinical and experimental level, oxidized low-density lipoprotein (oxLDL) in the diabetic state is immunogenic and can form oxLDL immune complexes, which activate the complement pathway via Fcγ receptor binding, induce the production of pro-inflammatory cytokines such as IL-1, IL-6, and TNF-α, and stimulate mesangial cells to secrete monocyte chemoattractant protein-1 (MCP-1) and collagen, thereby facilitating inflammatory cell recruitment and fibrosis (84). Furthermore, in basic T cell immunometabolism studies, Soroosh et al. demonstrated that the cholesterol derivative 7β,27-dihydroxycholesterol is a direct agonist of RORγt and drives Th17 differentiation, while Berod et al. showed that acetyl-CoA carboxylase 1 (ACC1), a key enzyme in fatty acid synthesis, acts as a molecular switch controlling the Th17/Treg balance: ACC1 inhibition promotes Treg differentiation and suppresses Th17 generation. These findings reveal that lipid metabolic reprogramming can directly determine CD4^+^ T cell lineage commitment at the molecular level; however, the specific roles and clinical significance of these regulatory pathways in the DKD renal microenvironment remain to be validated (85, 86). In summary, lipid metabolic disturbances impinge on CD4^+^ T cell function at multiple core nodes in DKD, yet direct causal evidence from T cell-specific genetic manipulations within the renal microenvironment is still lacking. Moreover, the synergistic or antagonistic interactions among different lipid pathways, as well as the lipid-mediated regulation of CD4^+^ T cell subsets beyond Th17/Treg, have been scarcely investigated. Addressing these gaps should represent a major focus for future research.

### 3.2 Key inflammatory pathways associated with CD4^+^ T cells in DKD

In the immunopathological progression of DKD, apart from metabolic factors, specific inflammatory signaling pathways play a crucial role by precisely regulating the activation, differentiation, and function of CD4^+^ T cells and their subsets. Notably, the programmed death-1 (PD-1)/programmed death ligand-1 (PD-L1) pathway, nuclear factor-κB (NF-κB) pathway, and molecules such as miR-155 and HSP70 play particularly critical roles. Collectively, these pathways shape the inflammatory landscape of DKD at multiple levels. Here, we discuss in detail the specific mechanisms by which these key pathways and molecules regulate the fate and function of CD4^+^ T cell subsets in DKD.

#### 3.2.1 The PD-1/PD-L1 pathway

Studies in DKD animal models have shown that the PD-1/PD-L1 pathway exerts renoprotective effects in DKD by modulating the Th17/Treg balance. Using an *in vitro* co-culture system of human placental mesenchymal stem cells (PMSCs) with lymphocytes stimulated by phytohemagglutinin and IL-2, Wang et al. demonstrated that PMSCs upregulate PD-1 expression in lymphocytes and downregulate their own PD-L1 expression in a cell-cell contact-dependent manner. PMSCs suppress excessive proliferation of activated CD4^+^ T cells, promote Treg cell differentiation, and inhibit Th17 cell activation. These changes result in significantly reduced levels of pro-inflammatory cytokines (IL-17A and IL-1β) and elevated levels of the anti-inflammatory cytokine IL-4, ultimately restoring the Th17/Treg balance and alleviating renal inflammatory injury (87). In the *in vivo* experiments of the study, transplantation of PMSCs into a rat model of STZ-induced DKD significantly improved renal function parameters and attenuated renal pathological injury, providing direct *in vivo* evidence for the renoprotective role of the PD-1/PD-L1 pathway in DKD (87). Furthermore, mass cytometry studies have revealed significantly elevated PD-1 expression in effector memory CD4^+^ T cells from the peripheral blood of patients with early DKD, suggesting that aberrant PD-1 expression may represent one of the characteristic features of immune dysregulation in DKD (88). However, these clinical findings currently remain largely at the level of correlative description. The molecular details of how the PD-1/PD-L1 pathway concretely regulates the fate and function of CD4^+^ T cell subsets within the renal microenvironment of DKD patients, and whether therapeutic strategies targeting this pathway can recapitulate the protective effects observed in animal models in a clinical setting, await further in-depth investigation.

#### 3.2.2 The NF-κB pathway

The NF-κB pathway serves as a critical upstream signaling axis linking CD4^+^ T cell activation to renal injury in DKD, and its aberrant activation markedly exacerbates renal inflammation (89, 90). Analysis of renal specimens from patients with DKD has demonstrated significantly enhanced activity of the NF-κB signaling pathway in diabetic kidney tissue (91). Southwestern histochemistry revealed that NF-κB is prominently activated primarily in cortical tubular epithelial cells, and its activation level is strongly positively correlated with the expression of MCP-1 and CCL5, as well as closely associated with the degree of proteinuria and interstitial cell infiltration (50). Mechanistically, activation of the NF-κB pathway not only enhances the activation capacity of CD4^+^ T cells, promoting their secretion of pro-inflammatory cytokines such as IL-17A and IFN-γ, but also induces renal resident cells to express chemokines including CCL2 and CCL5, thereby directionally recruiting CD4^+^ T cells to infiltrate renal tissue and amplifying local inflammatory responses. Notably, a “cross-activation” effect exists between IL-17A secreted by Th17 cells and the NF-κB pathway. In a mouse model of DKD, blockade of IL-17A signaling with a neutralizing antibody significantly suppressed NF-κB activation and the expression of downstream pro-inflammatory cytokines, attenuating renal inflammatory injury. Furthermore, animal experiments have also demonstrated that the NF-κB inhibitor NF-κB essential modulator-binding domain peptide can alleviate renal injury in DKD (90, 91). *In vitro*, stimulation of cultured podocytes with AGEs upregulated the expression of IL-17A, CD40, and TGF-β1, and promoted NF-κB activation; conversely, treatment with an anti-IL-17 monoclonal antibody reduced CD40 and TGF-β1 expression and increased podocyte viability, further confirming the role of the IL-17A–NF-κB feedback loop in podocyte injury (91). Through this bidirectional regulatory mechanism with CD4^+^ T cells, the NF-κB pathway not only amplifies the local inflammatory cascade in the kidney but, more importantly, reveals that intervention targeting this pathway may represent an effective strategy to break the inflammatory vicious cycle in DKD. This provides a theoretical basis for developing immunomodulatory therapies centered on NF-κB.

#### 3.2.3 Other regulatory molecules

Both regulatory molecules miR-155 and HSP70 influence the development and progression of DKD by modulating the Th17/Treg balance. *In vivo* and *in vitro* studies by Lin et al. have shown that miR-155 deficiency leads to significantly upregulated expression of suppressor of cytokine signaling 1 (SOCS1) in CD4^+^ T cells. As a negative regulator of the JAK/STAT pathway, SOCS1 can inhibit the activity of JAK kinases and reduce the phosphorylation levels of STAT3 and STAT5. Given that STAT3 serves as a key transcription factor for Th17 cell differentiation, reduced STAT3 activity directly suppresses Th17 cell differentiation and decreases IL-17A secretion, thereby modulating disease progression in DKD. This mechanism not only attenuates Th17-mediated inflammation but also promotes renin acetylation and alleviates podocyte injury induced by hyperglycemia, thus delaying the progression of DKD (22). On another regulatory axis, animal studies have elucidated the role of the HSP70/Tim-3 pathway in DKD. In a mouse model of type 2 DKD, highly expressed HSP70 in the kidney suppresses T-cell immunoglobulin and Tim-3—a surface molecule crucial for maintaining the anti-inflammatory function of Treg cells—thereby impairing Treg function, driving excessive Th17 cell activation, and increasing the secretion of pro-inflammatory cytokines. This results in a Th17/Treg imbalance, which ultimately amplifies local renal inflammation and accelerates the pathological progression of DKD. Conversely, inhibition of HSP70 upregulates Tim-3 expression, restores the Th17/Treg balance, and attenuates renal injury (92). In the immunoregulatory network of DKD, numerous other regulatory molecules participate in disease progression by influencing the balance of CD4^+^ T cell subsets. However, current understanding of this regulatory network remains incomplete, and the precise mechanisms underlying many potential molecules warrant further investigation.

### 3.3 Crosstalk of CD4^+^ T cells with renal immune and parenchymal cells and the amplification of inflammation

The crosstalk between CD4^+^ T cells and multiple immune cells as well as parenchymal cells within the diabetic kidney microenvironment represents one of the key mechanisms driving inflammatory amplification and accelerating renal injury in DKD. These cells include Mø, dendritic cells (DCs), group 2 innate lymphoid cells (ILC2s), RTCs, and B cells (73, 93). Among them, Mø, DCs, and ILC2s serve as pivotal bridging cells between innate and adaptive immunity, exerting an initiating trigger role in immune activation during DKD (94, 95). Specifically, in the setting of DKD, pathological signals such as hyperglycemia, hypoxia, and oxidative stress in the local kidney are sensed by Mø and DCs, driving their activation and subsequent secretion of pro-inflammatory cytokines including IL-1β, IL-6, and TNF-α. As professional antigen-presenting cells, DCs prime naïve CD4^+^ T cells via processing and presenting kidney damage-associated antigens. In contrast, ILC2s modulate the differentiation trajectory of CD4^+^ T cells by secreting cytokines such as IL-4 and IL-13. Under the synergistic effects of these three cell types, naïve CD4^+^ T cells are directionally polarized into pro-inflammatory subsets including Th1 and Th17 cells. Activated effector Th cells further secrete pro-inflammatory mediators like IFN-γ and IL-17A, which not only directly exacerbate inflammatory injury in renal resident cells but also promote chemokine expression to recruit more immune cells (e.g., Mø and DCs) into the kidney, thereby forming a vicious cycle of inflammatory amplification. As another core component of adaptive immunity, B cells not only promote local immune activation by forming renal ectopic tertiary lymphoid structures in coordination with Tfh cells, but also reshape the polarization landscape of CD4^+^ T cells through imbalances among distinct B cell subsets, thereby exerting a bidirectional regulatory role in the immunopathology of DKD (**Figure 4**).

**Figure 4:**
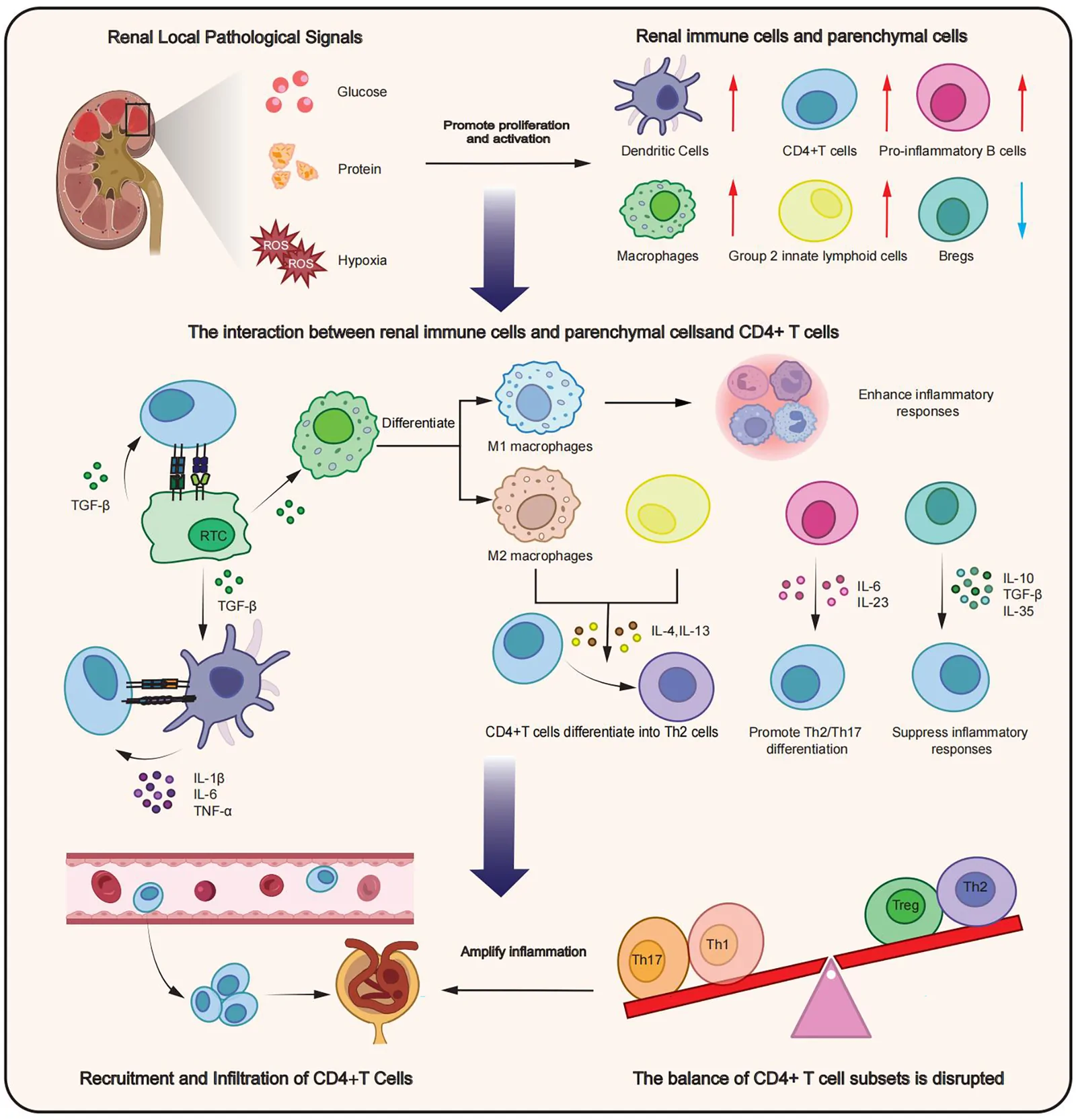
Bidirectional crosstalk between renal resident parenchymal and immune cells and CD4^+^ T cells that amplifies inflammatory cascades and accelerates renal fibrosis in DKD. Pathological stimuli including hyperglycemia, proteinuria, reactive oxygen species and hypoxia activate multiple intrarenal resident cells to form a complex immune regulatory network with CD4^+^ T cells. Dendritic cells recognize renal damage signals via toll-like receptors and NOD-like receptors, activate NLR family pyrin domain-containing 3 inflammasome and nuclear factor-κB (NF-κB) signaling, and present autoantigens to naive CD4^+^ T cells to drive their differentiation into pro-inflammatory Th1 and Th17 subsets. Macrophages exhibit functional plasticity: Th1-derived IFN-γ and IL-17A recruit monocytes and promote M1 polarization to secrete pro-inflammatory mediators, whereas anti-inflammatory M2 macrophages induce Th2 and Treg differentiation through paracrine signals and cell contact. Stimulated renal tubular epithelial cells secrete heat shock protein 70 and chemokines, and express major histocompatibility complex class II to directly trigger CD4^+^ T cell proliferation and Th2 skewing. Group 2 innate lymphoid cells activated by renal injury release IL-4, IL-5 and IL-13 to facilitate Th2 differentiation and counteract Th1 responses. Ectopic B cell-rich tertiary lymphoid structures emerge in DKD renal interstitium, where the CXCL13–CXCR5 axis supports the co-localization of B cells and Tfh cells; pro-inflammatory IgD^+^CD38^+^ naive B cells secrete IL-6 and IL-23 to boost Th2 and Th17 responses, while regulatory B cells produce IL-10, TGF-β and IL-35 to inhibit effector T cells and expand Tregs. This integrated cell-cell communication network disrupts the balanced distribution of CD4^+^ T subsets, recruits massive pro-inflammatory T cell infiltration, and sustains self-amplifying intrarenal inflammation that ultimately drives progressive glomerulosclerosis and tubulointerstitial fibrosis. DKD, diabetic kidney disease; ROS, reactive oxygen species; DCs, dendritic cells; TLRs, toll-like receptors; NLRs, NOD-like receptors; NLRP3, NOD-like receptor pyrin domain-containing 3; NF-κB, nuclear factor-κB; Th1, T helper type 1; Th17, T helper type 17; Mø, macrophages; IFN-γ, interferon-γ; IL, interleukin; RTCs, renal tubular epithelial cells; HSP70, heat shock protein 70; MHC-II, major histocompatibility complex class II; ILC2s, group 2 innate lymphoid cells; Tfh, T follicular helper cell; Bregs, regulatory B cells; TGF-β, transforming growth factor-β.

#### 3.3.1 DCs

Current evidence regarding the function of DCs in the renal immune microenvironment encompasses multiple levels, including histological observations in humans, *in vivo* animal experiments, and *in vitro* cell studies. Histological evidence from human renal tissues indicates that DCs, as key antigen-presenting cells, are widely distributed in the tubulointerstitial compartment; animal model studies have further demonstrated that, by executing a cascade of sensing damage signals, activating immune pathways, and recruiting and activating CD4^+^ T cells, DCs serve as a central hub that links innate and adaptive immunity and drives the progression of renal inflammation. *In vitro* cell experiments have shown that DCs sense exogenous pathogens and endogenous damage signals derived from injured or necrotic renal tubular cells via pattern recognition receptors expressed on their surface and in the cytoplasm, including TLRs and NOD-like receptors (NLRs). This recognition subsequently activates the NOD-like receptor family pyrin domain containing 3 (NLRP3) inflammasome, which modulates transcription factors such as IRF7 and NF-κB to orchestrate chemokine secretion and recruit immune cells including CD4^+^ T cells. Furthermore, both *in vitro* antigen presentation co-culture experiments and *in vivo* cell tracking studies have demonstrated that CD4^+^ T cells migrating into proximity with DCs can be activated via TCR signaling, thereby driving a specific adaptive immune response (96–98).

#### 3.3.2 Mø

Mø are among the most abundantly infiltrated immune cells in the kidneys of patients with DKD. Single-cell transcriptomic sequencing studies have revealed that Mø are the predominant infiltrating immune cells in the kidney during early DKD, and the extent of their infiltration is significantly positively correlated with the rate of renal function decline as well as the severity of glomerulosclerosis and tubulointerstitial fibrosis, suggesting that they play a central driving role in the initiation and progression of the disease (99–101). Based on their developmental origin, Mø can be categorized into kidney-resident Mø and monocyte-derived Mø (102). In animal studies, DKD mouse models have demonstrated that resident Mø display enhanced ROS production and upregulated phagocytic activity (103). With respect to monocyte-derived Mø, DKD models have shown that they are recruited and undergo differentiation under the stimulation of high glucose and AGEs (103). Single-cell sequencing of an AKI-to-CKD mouse model further revealed that monocyte-derived ECM-remodeling-associated Mø promote CD4^+^ T cell differentiation toward Th1/Th17 phenotypes by secreting pro-inflammatory cytokines such as TNF-α (104). However, these conclusions are largely drawn from animal and cell-based experiments. In human kidney biopsy tissues, only co-localization of Mø and T cells has been observed to date, and direct evidence regarding the dynamics and functional interactions of Mø subsets remains absent. Consequently, clinical translation still requires in-depth validation in DKD patient samples leveraging single-cell technologies.

Mø exhibit remarkable plasticity within the renal microenvironment of DKD. They can differentiate into two functionally distinct subsets, pro-inflammatory M1ø or anti-inflammatory M2ø, via distinct activation pathways. This plasticity in polarization status renders them key players in the pathogenesis of DKD (105, 106). M1ø are induced by pathogen-associated molecular patterns, damage-associated molecular patterns, IL-1α, and TNF-α. They highly express effector molecules such as IL-1, IL-6, inducible nitric oxide synthase, and matrix metalloproteinase-12. By inhibiting *ABCA1/ABCG1* gene expression, they impair cholesterol efflux and promote foam cell formation. Moreover, their interaction with CD4^+^ T cells generates various reactive oxygen species, pro-inflammatory cytokines, metalloproteinases, and growth factors, thereby amplifying local immune responses and exacerbating intrarenal inflammation in DKD (102, 107, 108). M2ø, in contrast, can be induced by IL-4, IL-13, or immune complexes and can also arise from the phenotypic conversion of M1ø. They are characterized by high expression of CD206, CD163, and arginase-1. Through cell–cell contact or paracrine mechanisms, M2ø promote the differentiation of CD4^+^ T cells toward the Th2 phenotype and enhance the expression of Th2-type cytokines, thereby primarily exerting anti-inflammatory and tissue repair functions (65). Based on differences in inducing conditions and functional phenotypes, M2ø can be further subdivided into four subsets: M2a, M2b, M2c, and M2d. M2a are mainly involved in wound healing and tissue fibrosis; M2b cells are predominantly responsible for immunoregulation; M2c mediate immunosuppression, matrix remodeling, and tissue repair through the secretion of IL-10 and matrix metalloproteinase-7; and M2d are more commonly found in the tumor microenvironment, with their specific function in DKD yet to be elucidated (109). Chronic hyperglycemia impairs the anti-inflammatory and reparative capacity of M2ø, resulting in an M1/M2 polarization imbalance that serves as a central mechanism driving the progression of chronic inflammation in DKD (109).

The polarization status of renal Mø is tightly regulated by upstream CD4^+^ T cell subsets. Animal studies have demonstrated that IFN-γ secreted by Th1 cells upregulates the expression of adhesion molecules on endothelial cells, while IL-17 produced by Th17 cells induces MCP-1 release, together promoting Mø recruitment into renal tissue and their polarization toward the M1 phenotype. In contrast, Treg cells negatively regulate Mø activity through the secretion of IL-10 and TGF-β, thereby exerting renoprotective effects (110, 111). These findings suggest that the above T cell–Mø interaction axes may also operate in human DKD; however, the dynamic changes in Mø subsets and their functional interplay with T cells still require in-depth validation in DKD clinical samples using single-cell technologies. Given the plastic polarization of Mø, future immunotherapeutic strategies for DKD may aim to modulate Mø polarization balance, rather than simply inhibiting or activating a specific subset. This provides a novel perspective for developing more precise immune interventions.

#### 3.3.3 RTCs

Although renal tubular cells are not immune cells per se, they can indirectly participate in and regulate local renal immune-inflammatory responses through metabolic disturbance–mediated alterations in the microenvironment and activation of cellular signaling pathways, thereby emerging as one of the key regulatory units in the immunopathogenesis of DKD. *In vitro* experiments have shown that high glucose and albumin stimulation induce RTCs to release HSP70 and activate NF-κB, thereby upregulating the expression of CCL2 and vascular cell adhesion molecule-1 (112, 113). Animal models have further confirmed that activation of the HSP70–TLR4 axis in the renal tubules of diabetic mice exacerbates tubular injury and interstitial inflammation. In fibrotic mice, proximal tubular epithelial cells present antigens via MHC class II molecules, directly promoting CD4^+^ T cell proliferation and Th2 differentiation, whereas tubule-specific knockout of *H2-Ab1* increases the proportion of Treg cells and attenuates fibrosis (114). Human biopsy evidence likewise shows significantly upregulated expression of TLR4, HSP70, and major histocompatibility complex class II (MHC-II) in the renal tubules of patients with DKD (114). The above conclusions regarding RTCs are mainly derived from animal and cell-based experiments; the dynamic process whereby RTCs directly activate CD4^+^ T cells through MHC-II in human DKD still requires in-depth validation using single-cell technologies in patient samples.

#### 3.3.4 ILC2

As a critical component of innate immunity, ILC2s contribute to the progression of DKD via the sequential cascade of “aberrant expansion–activation upon stimulation–modulation of CD4^+^ T cell differentiation–modulation of inflammation and fibrosis”. Baeten et al. reported in a clinical trial that the proportion of ILC2s and the levels of IL-4, IL-5, and IL-13 were significantly elevated in the peripheral blood of patients with DKD, and further increased with the severity of DKD. These findings suggest that aberrant ILC2 abundance may serve as an important marker of immune dysregulation in DKD and is closely associated with the progression of renal injury (40, 95). Pathological signals such as hyperglycemia and renal cell damage activate ILC2s via the induction of specific cytokines. Activated ILC2s directly regulate fibrosis by secreting Th2-type cytokines and further drive the differentiation of CD4^+^ T cells toward the Th2 subset; meanwhile, they suppress Th1 differentiation through contact-dependent mechanisms. Together, these two mechanisms render ILC2s a critical mediator in regulating the Th1/Th2 balance (40, 92, 115, 116).

#### 3.3.5 B cells

In addition to Mø and DCs, B cells also represent a crucial link between innate and adaptive immunity and actively participate in the immunopathological network of DKD. A recent spatial transcriptomics study has confirmed that ectopic B cell-rich tertiary lymphoid structures can form within the renal tissue of some DKD patients, and CD4^+^ T cells within these structures highly express a Tfh phenotype (117). Within this specialized microenvironment, the CXCL13–CXCR5 signaling axis is prominently co-expressed, mediating the directed co-localization and local aggregation of B cells and Tfh cells. Concurrently, aberrant activation of the B-cell activating factor–BAFF receptor signaling pathway provides essential support for the local survival and differentiation of B cells. Infiltrating B cells highly express activation- and differentiation-associated molecules such as activation-induced cytidine deaminase and IRF4, and display Ki-67 positivity indicative of proliferative activation, suggesting that Tfh cells *in situ* can assist B cells in completing a series of immune responses, including activation, proliferation, and antibody class switching.

Different B cell subsets can differentially regulate CD4^+^ T cell polarization through the secretion of specific cytokines, thereby reshaping the local renal immune microenvironment. Clinical and omics studies have confirmed that, compared with healthy controls, the proportions of pro-inflammatory and activated B cell subsets—including plasma cells, activated B cells, and IgD^+^CD38^+^ activated naïve B cells—are significantly increased in the kidney tissue and peripheral blood of DKD patients, whereas the numbers of immunosuppressive regulatory B cells (Bregs) are markedly reduced, culminating in an imbalanced B cell subset landscape (117–119). This imbalance mirrors the CD4^+^ T cell-mediated Th17/Treg immune dysregulation and jointly drives persistent chronic renal inflammation and fibrosis. Specifically, IgD^+^CD38^+^ activated naïve B cells may promote the conversion of CD4^+^ T cells into Th2 and Th17 effector cells by secreting pro-inflammatory cytokines such as IL-6 and IL-23, thereby exacerbating local Th2-type and Th17-type inflammatory responses. In contrast, Breg subsets can produce inhibitory cytokines, including IL-10, TGF-β, and IL-35, which on the one hand directly suppress the pro-inflammatory activation of Th1 and Th17 cells and block the inflammatory cascade, and on the other hand induce the differentiation and expansion of Treg cells to maintain immune homeostasis and exert renoprotective effects (119). Furthermore, Mendelian randomization studies have indicated that functional markers of IgD^+^CD27^+^ memory B cells are inversely causally associated with the risk of DKD, suggesting that this memory B cell subset may participate in protective renal immune regulation and delay the onset and progression of DKD (119). At present, research on the crosstalk between B cells and CD4^+^ T cells in DKD remains relatively limited. The specific molecular pathways, spatiotemporal regulatory characteristics, and clinical translational value of how distinct B cell subsets mediate T cell polarization and contribute to renal inflammation and fibrosis have yet to be fully elucidated, and further in-depth validation through large-scale clinical cohort studies and basic mechanistic experiments is warranted.

## 4 CD4^+^ T cell-targeted therapeutic strategies in DKD

Current clinical management of DKD remains centered on renin–angiotensin system blockade, primarily achieved with angiotensin-converting enzyme inhibitors or angiotensin II receptor blockers. Meanwhile, novel agents represented by SGLT2i have provided additional options for improving patient prognosis (120). With advancing insights into the immunopathogenic mechanisms of DKD, targeted therapeutic strategies against CD4^+^ T cells and their secreted factors have garnered considerable attention, as they directly act on key checkpoints governing disease progression (**Table 1**). Building on this foundation, the following section highlights several representative agents and therapeutic approaches, with a focus on elaborating their pharmacological mechanisms underlying the regulation of CD4^+^ T cell subset balance and the latest research advances.

**Table 1:** Summary of representative therapeutic interventions targeting CD4^+^ T cell subsets for DKD treatment, alongside their immunomodulatory mechanisms, experimental models, primary renal protective endpoints, CD4^+^ T cell-related effects, and translational limitations.

Drug name	Drug category	Mechanism of action	Experimental model/population	Principal renal endpoint	CD4^+^ T-cell endpoint	Limitations	References
Metformin	Antidiabetic agents	Inhibits NF-κB pathway and MHC class II expression, reduces CD4^+^ T cell infiltration and Th1/Th17 skewing, impairs DC antigen presentation.	STZ + high-fat diet mouse model of DKD; db/db mice; *in vitro* DC culture.	Reduced Scr, BUN, UAlb; alleviated renal fibrosis.	Decreased renal CD4^+^ T infiltration and IFN-γ secretion; downregulated renal MHC II (*H2-Aa*, *H2-Ab1*).	Direct human DKD clinical evidence for CD4^+^ T modulation is absent; mechanistic data rely on animal studies.	(122–124)
Liraglutide	Antidiabetic agents	Blocks NLRP3/NF-κB and TGF-β1/Smad cascades; suppresses CD4^+^ T proliferation and Th1/Th17 skewing via inhibiting T cell glycolysis and Glut1 expression.	T-cell-dependent NTN murine model; diabetic rodents; type 2 diabetes (T2D) clinical cohorts.	Reduced incident macroalbuminuria; attenuated renal fibrosis.	Inhibited CD4^+^ T proliferation and Th1/Th17 differentiation.	Definitive causal evidence of direct intrarenal CD4^+^ T modulation in human DKD remains unvalidated.	(125, 126)
Triptolide	TCM herbs	Restores Th1/Th2 balance (↓Th1, ↑Th2); suppresses NF-κB activation and Mø infiltration.	Meta-analysis of DKD rodent models; STZ-induced DKD rats.	Reduced UAlb; preserved global renal function.	Corrected intrarenal Th1/Th2 imbalance; suppressed renal NF-κB signaling.	Severe multi-organ toxicity and narrow therapeutic window hinder clinical translation.	(29, 99)
Hyperoside	TCM herbs	Promotes M1ø to M2ø phenotypic shift; inhibits CD4^+^ T proliferation and facilitates Th2/Treg differentiation.	T2D murine DKD; *in vitro* M2ø-CD4^+^ T co-culture.	Mitigated renal inflammatory injury and TIF.	Suppressed CD4^+^ T overproliferation; boosted Th2 and Treg differentiation.	No dedicated human DKD clinical trials; pharmacokinetic profiles uncharacterized.	(59)
Mycophenolate mofetil	Th17-targeted agents	Blunts Th17 differentiation and IL-17-related chemokine release; reduces intrarenal Mø and CD4^+^ T infiltration.	Multiple murine DKD models.	Attenuated tubulointerstitial inflammation and fibrosis.	Inhibited Th17 lineage commitment; lowered intrarenal IL-17 levels.	Scarce human DKD trial data; long-term systemic immunosuppression carries safety risks.	(42, 100)
IL-17A neutralizing antibody	Th17-targeted agents	Specifically binds and neutralizes IL-17A to block downstream pro-inflammatory signaling cascades.	STZ and db/db murine DKD models.	Improved renal structural and functional lesions; suppressed renal injury biomarkers.	Complete blockade of IL-17A-mediated Th17 effector function.	All available data derive from preclinical animal work; single-cytokine blockade cannot reverse full CD4^+^ T subset imbalance.	(42, 47)
Finerenone	Nonsteroidal MRA	Blocks Aldo-MR axis to suppress aberrant Th1/Th17 activation and expand renal Treg populations.	Preclinical DKD rodents; human T2D-DKD cohorts.	Delayed DKD progression; alleviated intrarenal inflammatory lesions.	Suppressed Th1/Th17 overactivation; preserved/expanded renal Treg abundance.	Landmark clinical trials lack direct quantitative CD4^+^ T subset monitoring data; mechanisms mainly preclinical.	(101–103)
Dapagliflozin	SGLT2i	Inhibits renal SGLT2-mediated sodium reabsorption; downregulates renal SGK1/p-FoxO1/IL-23R axis to reverse Th17/Treg imbalance.	High-salt db/db murine DKD; human T2D cohorts.	Reduced UACR; reversed TIF.	Restored systemic and intrarenal Th17/Treg equilibrium; suppressed renal SGK1 signaling.	T cell immunomodulatory effects only validated in animal studies; human immune longitudinal monitoring missing.	(43, 50)
PMSCs	Cell-based therapy	Restores Th17/Treg balance via PD-1/PD-L1-dependent suppression of excessive CD4^+^ T proliferation.	STZ-induced DKD rat model; *in vitro* lymphocyte co-culture.	Ameliorated renal pathological damage and improved renal function metrics.	Reduced Th17 proportion, elevated Treg abundance; lowered intrarenal IL-17A, elevated IL-4.	Entirely preclinical evidence; long-term safety and efficacy in human subjects unconfirmed.	(75)

All abbreviations are defined at the bottom of the table.

DKD, diabetic kidney disease; STZ, streptozotocin; Scr, serum creatinine; BUN, blood urea nitrogen; UAlb, urinary albumin; UACR, urinary albumin-creatinine ratio; TIF, tubulointerstitial fibrosis; NF-κB, nuclear factor-κB; MHC-II, major histocompatibility complex class II; DC, dendritic cell; NLRP3, NOD-like receptor pyrin domain-containing 3; TGF-β1, transforming growth factor-β1; Glut1, glucose transporter 1; NTN, nephrotoxic serum nephritis; T2D, type 2 diabetes mellitus; TCM, traditional Chinese medicine; Mø, macrophage; M1/M2ø, M1/M2 macrophage; IL, interleukin; IFN-γ, interferon-γ; Nonsteroidal MRA, nonsteroidal mineralocorticoid receptor antagonist; Aldo-MR, aldosterone-mineralocorticoid receptor; SGLT2i, sodium-glucose cotransporter 2 inhibitor; SGK1, serum/glucocorticoid-regulated kinase 1; p-FoxO1, phosphorylated forkhead box O1; PMSCs, placental mesenchymal stem cells; PD-1/PD-L1, programmed death-1/programmed death ligand-1.

### 4.1 Common clinical antihyperglycemic agents

Metformin, as a first-line agent for T2D, has well-recognized renoprotective effects. Beyond improving glucose metabolism and insulin resistance, recent studies have revealed that metformin can directly participate in the regulation of the local renal immune microenvironment, with a particular relevance to CD4^+^ T cell-mediated adaptive immune responses. In a mouse model of DKD induced by STZ combined with a high-fat diet, metformin treatment significantly decreased serum creatinine, blood urea nitrogen, and urinary albumin, accompanied by reduced circulating levels of the pro-inflammatory cytokines IL-6, TNF-α, and IL-1β and the chemokine MCP-1, as well as suppressed renal oxidative stress and NF-κB pathway activation (121). These effects suggest that metformin may indirectly attenuate the local inflammatory milieu that drives CD4^+^ T cell polarization toward Th1/Th17 phenotypes. More direct evidence comes from the MHC class II pathway. In db/db mice, the combination of metformin and Jin-Gui-Shen-Qi Wan synergistically downregulated the expression of the MHC-II molecules *H2-Aa* and *H2-Ab1* in the kidney, reduced CD4^+^ T cell infiltration and the release of the Th1 effector cytokine IFN-γ, and consequently alleviated renal fibrosis (122). It is known that metformin can inhibit the expression of MHC-II molecules and the co-stimulatory molecules CD80/CD86 on the surface of dendritic cells, thereby impairing their capacity for antigen presentation to CD4^+^ T cells (123).

Direct evidence for the regulation of CD4^+^ T cells by GLP-1RAs in DKD remains limited; however, available studies suggest that GLP-1RAs may exert immunomodulatory effects through multiple mechanisms. As a prototypical GLP-1RA, liraglutide has been shown in diabetic models to inhibit renal NLRP3 inflammasome activation and the NF-κB pathway, reduce the levels of pro-inflammatory cytokines such as IL-1β, IL-6, and TNF-α and the chemokine MCP-1, and simultaneously attenuate renal fibrosis by downregulating TGF-β1/Smad signaling (124). However, whether these alterations in downstream cytokines and fibrotic pathways are mediated by a direct action of GLP-1RAs on renal CD4^+^ T cells remains to be established with definitive causal experimental evidence. At the level of T-cell immune regulation, liraglutide has been demonstrated in a T-cell-dependent nephrotoxic serum nephritis model to directly suppress CD4^+^ T cell proliferation and Th1/Th17 polarization through mechanisms involving the blockade of T-cell glycolysis and downregulation of the glucose transporter Glut1. Notably, that study explicitly indicates that this glucose-lowering-independent inhibition of T cells may partly explain the benefit of GLP-1RAs in reducing new-onset macroalbuminuria in patients with T2D observed in large-scale clinical trials (125). Collectively, the immunoprotective effects of GLP-1RAs in DKD may involve multiple mechanisms, including the inhibition of CD4^+^ T cell proliferation and effector function, the downregulation of the renal pro-inflammatory cytokine network, and the attenuation of fibrosis; however, the direct roles of these immunomodulatory actions within the DKD renal microenvironment and their clinical translational value still require further in-depth investigation.

### 4.2 Traditional Chinese herbal medicines

Triptolide (TP), a diterpenoid triepoxide and the major bioactive constituent of *Tripterygium wilfordii* Hook.f. Meta-analyses of animal studies have demonstrated that TP significantly reduces albuminuria and preserves renal function in DKD (126). Animal studies have shown that TP is capable of regulating the Th1/Th2 cell balance, manifested by downregulating the proportion of Th1 cells and the release of related pro-inflammatory cytokines, while upregulating the proportion of Th2 cells and the expression of associated anti-inflammatory cytokines. Furthermore, TP can ameliorate renal injury in DKD by inhibiting NF-κB pathway activation and Mø infiltration, thereby reducing the release of inflammatory and fibrotic factors (30). Despite these promising preclinical effects, the clinical translation of TP is limited by its multi-organ toxicity and narrow therapeutic window, including hepatotoxicity, nephrotoxicity, and reproductive toxicity (127, 128). Current translational strategies to address these challenges involve the development of kidney-targeted delivery systems (e.g., TP-encapsulated mesoscale nanoparticles) and less toxic structural analogs (e.g., triptolide aminoglycoside, TPAG), which have shown improved safety profiles in preclinical studies (129, 130).

Hyperoside (HPS), chemically quercetin 3-O-β-D-galactopyranoside and the principal bioactive constituent isolated from *Cuscuta chinensis* Lam., has also been demonstrated to confer renoprotective effects in the setting of DKD. In a co-culture system of M2ø and CD4^+^ T cells, HPS directly or indirectly suppresses the proliferation of CD4^+^ T cells and drives their differentiation toward Th2 and Treg cell subsets, thereby mediating renoprotective effects (65). In addition, *in vivo* animal studies have demonstrated that HPS promotes the transition of M1ø to the M2 phenotype, thereby inhibiting the infiltration of pro-inflammatory Mø in the kidney, reducing the expression of pro-inflammatory cytokines, and increasing the production of anti-inflammatory cytokines. *In vitro* cell culture experiments have further confirmed that HPS can directly induce bone marrow-derived Mø to polarize into the M2 phenotype (65). Unlike TP, HPS exhibits high efficacy and low toxicity in preclinical models (65). However, its clinical translation remains limited by the absence of DKD-specific trials and unestablished pharmacokinetics.

### 4.3 Agents targeting Th17 cells

Targeting Th17 cells and their effector molecule IL-17A represents a major research focus. Mycophenolate mofetil (MMF) and IL-17A neutralizing antibodies have been shown to attenuate renal injury in DKD by modulating Th17 cell function. MMF is a widely used immunosuppressive agent in clinical practice, which exerts multi-faceted renoprotective effects in DKD independently of glycemic control (50). Its mechanisms of action extend beyond inhibiting Th17 cell differentiation and reducing the production of IL-17–associated chemokines. MMF also mitigates macrophage and CD4^+^ T-cell infiltration, downregulates the expression of pro-inflammatory cytokines such as IL-17 and TNF-α, and thereby alleviates renal tissue injury caused by inflammatory responses (45, 131). IL-17A neutralizing antibodies exert their effects by specifically binding to IL-17A and blocking its biological activity, thereby serving as a precise intervention strategy targeting Th17 cells. These antibodies have been shown to ameliorate renal functional and structural damage and significantly downregulate the expression of renal injury markers (45, 50).

DKD is a chronic immune-inflammatory disease mediated by the imbalance of CD4^+^ T cell subsets. Aberrant activation of Th17 cells is not the sole pathogenic factor; Th1, Th2, Treg subsets and their relevant effector cytokines all participate in disease progression. Building on the therapeutic efficacy of Th17-targeted agents, exploring neutralizing antibodies and immunomodulators targeting Th1, Th2 subsets and their signature inflammatory cytokines represents a promising novel direction for DKD immunotherapy. A study in patients with T2D showed that serum levels of JNK pathway-associated phosphatase (JKAP) are positively correlated with the proportion of Th2 cells and negatively correlated with the proportion of Th17 cells, and that JKAP is an independent protective factor against the risk and severity of DKD, suggesting its potential as a therapeutic target (21). Animal experiments further revealed that JKAP can dephosphorylate Lck at tyrosine 394, thereby inhibiting T cell receptor signaling and negatively regulating T cell-mediated immune responses and autoimmunity (132). However, targeted interventions against Th1 and Th2 subsets remain largely unexplored at present. Key issues including cellular crosstalk and target safety urgently need to be addressed. Future research should further elucidate the regulatory mechanisms of T-cell subsets, advance target validation and drug development, and facilitate precision immunotherapy for DKD.

### 4.4 Nonsteroidal mineralocorticoid receptor antagonists

Finerenone is a novel nonsteroidal mineralocorticoid receptor (MR) antagonist. Compared with the first-generation steroidal MR antagonists spironolactone and the second-generation agent eplerenone, finerenone exhibits higher affinity for MR expressed in T cells and fewer adverse effects, and more effectively inhibits the recruitment of transcriptional coactivators involved in the expression of pathogenic genes (133, 134). Excessive activation of the aldosterone–MR axis promotes the differentiation of naive CD4^+^ T cells into pro-inflammatory subsets such as Th1 and Th17 cells, reduces the number of Treg cells, and exacerbates the imbalance between pro-inflammatory and anti-inflammatory responses in the kidney. By blocking the aldosterone–MR axis, finerenone markedly suppresses the abnormal activation of Th1 and Th17 cells while preserving or increasing the proportion of Treg cells, thereby mitigating CD4^+^ T cell-mediated renal inflammatory injury and delaying the progression of DKD (135).

### 4.5 SGLT2i

Dapagliflozin (Dap), a representative SGLT2i, not only regulates glucose metabolism via the classic glucosuric and diuretic mechanisms but also exerts specific renoprotective effects in DKD by modulating the balance of CD4^+^ T cell subsets and improving the renal pathological microenvironment. In a mouse model of DKD, Dap inhibits the expression of SGK1, p-FoxO1, and IL-23R in Th17 cells within renal tissues of DKD (46). Furthermore, by attenuating SGLT2-mediated local high-salt conditions in the microenvironment, Dap restores the imbalance between Th17 and Treg cells in both the renal and peripheral immune microenvironments of DKD mice, reduces albuminuria, and reverses tubulointerstitial fibrosis, ultimately conferring renal protection in DKD mice (55).

### 4.6 PMSCs

PMSCs represent a promising novel strategy and fundamental basis for the future clinical treatment of DKD. In a rat model of streptozotocin-induced DKD, administration of PMSCs for 16 weeks markedly improved renal function and alleviated renal pathological injury. These effects were accompanied by a decreased proportion of Th17 cells and an increased proportion of Treg cells in the peripheral blood and spleen, as well as reduced levels of pro-inflammatory cytokines IL-17A and IL-1β and elevated levels of the anti-inflammatory cytokine IL-4 in the kidney and serum. *In vitro* studies further confirmed that PMSCs regulate the Th17/Treg balance via the PD-1/PD-L1 signaling pathway (87).

## 5 Summary and perspectives

This review provides a comprehensive analysis of the central pathogenic and regulatory mechanisms of CD4^+^ T-cell subsets in the progression of DKD. Accumulating evidence clearly delineates that chemotactic infiltration and imbalanced polarization of CD4^+^ T cells—characterized by skewed ratios of Th1/Th2 and Th17/Treg—constitute the pivotal trigger for renal inflammation and fibrosis in DKD. These subsets secrete a panel of effector molecules, establishing intimate crosstalk with both the systemic metabolic microenvironment and renal resident cells, thereby constructing an intricate network underlying immune-mediated renal pathogenesis in DKD. Nevertheless, this only represents the tip of the iceberg. The precise roles and pathological contributions of understudied subsets, including Th3, Th9, Th22 and Tfh cells, in DKD remain largely uncharacterized, representing a critical knowledge gap that urgently needs to be addressed using cutting-edge techniques such as high-throughput single-cell sequencing (136–139).

Nevertheless, profound challenges remain in advancing this field to greater depths. First, current mechanistic conclusions rely heavily on rodent models, and there exists an irreconcilable species gap between their immune landscapes and the true microenvironment of human DKD. Second, although the primary impacts of glucolipotoxicity and oxidative stress have been established, the deeper mechanisms governing metabolic–immune crosstalk remain poorly defined. For instance, how dysregulated metabolites (e.g., lactate, ketone bodies, uric acid) and electrolyte imbalance reshape CD4^+^ T-cell fate via epigenetic pathways remains largely elusive. Furthermore, translating targeted immunotherapies into clinical practice requires overcoming major barriers related to drug safety, long-term immune tolerance, and high patient heterogeneity.

Looking ahead, research into DKD pathogenesis must undergo a paradigm shift from “single-pathway” investigations to “spatiotemporal multi-omics” at a holistic level. Only by comprehensively decoding the authentic immune microenvironment of human DKD and delineating the dynamic interplay between metabolic flux, epigenetic regulation, and immune reprogramming can we break through the current bottleneck in understanding. Building on this foundation, the development of innovative therapies featuring precise targeting and individualized modulation of CD4^+^ T cells will undoubtedly shed new light on reversing the course of DKD and improving long-term patient outcomes.
